# Exploring morphological similarity and randomness in Alzheimer’s disease using adjacent grey matter voxel-based structural analysis

**DOI:** 10.1186/s13195-024-01448-1

**Published:** 2024-04-23

**Authors:** Ting-Yu Chen, Jun-Ding Zhu, Shih-Jen Tsai, Albert C. Yang

**Affiliations:** 1 Institute of Brain Science, National Yang Ming Chiao Tung University, Taipei, Taiwan; 2 Digital Medicine and Smart Healthcare Research Center, National Yang Ming Chiao Tung University, Taipei, Taiwan; 3 Department of Psychiatry, Taipei Veterans General Hospital, Taipei, Taiwan; 4 Division of Psychiatry, School of Medicine, National Yang Ming Chiao Tung University, Taipei, Taiwan; 5 Department of Medical Research, Taipei Veterans General Hospital, Taipei, Taiwan; 6 Brain Research Center, National Yang Ming Chiao Tung University, Taipei, Taiwan

**Keywords:** Alzheimer’s disease, Morphological similarity network, Structural magnetic resonance imaging, Information-based similarity method

## Abstract

**Background:**

Alzheimer’s disease is characterized by large-scale structural changes in a specific pattern. Recent studies developed morphological similarity networks constructed by brain regions similar in structural features to represent brain structural organization. However, few studies have used local morphological properties to explore inter-regional structural similarity in Alzheimer’s disease.

**Methods:**

Here, we sourced T1-weighted MRI images of 342 cognitively normal participants and 276 individuals with Alzheimer’s disease from the Alzheimer's Disease Neuroimaging Initiative database. The relationships of grey matter intensity between adjacent voxels were defined and converted to the structural pattern indices. We conducted the information-based similarity method to evaluate the structural similarity of structural pattern organization between brain regions. Besides, we examined the structural randomness on brain regions. Finally, the relationship between the structural randomness and cognitive performance of individuals with Alzheimer’s disease was assessed by stepwise regression.

**Results:**

Compared to cognitively normal participants, individuals with Alzheimer’s disease showed significant structural pattern changes in the bilateral posterior cingulate gyrus, hippocampus, and olfactory cortex. Additionally, individuals with Alzheimer’s disease showed that the bilateral insula had decreased inter-regional structural similarity with frontal regions, while the bilateral hippocampus had increased inter-regional structural similarity with temporal and subcortical regions. For the structural randomness, we found significant decreases in the temporal and subcortical areas and significant increases in the occipital and frontal regions. The regression analysis showed that the structural randomness of five brain regions was correlated with the Mini-Mental State Examination scores of individuals with Alzheimer’s disease.

**Conclusions:**

Our study suggested that individuals with Alzheimer’s disease alter micro-structural patterns and morphological similarity with the insula and hippocampus. Structural randomness of individuals with Alzheimer’s disease changed in temporal, frontal, and occipital brain regions. Morphological similarity and randomness provide valuable insight into brain structural organization in Alzheimer’s disease.

**Supplementary Information:**

The online version contains supplementary material available at 10.1186/s13195-024-01448-1.

## Background

Alzheimer’s disease (AD) is a progressive neurodegenerative disease and the most common cause of dementia. Individuals with AD have brain structural alterations related to amyloid-β plaques and tau-related neurofibrillary tangles including atrophy in the cortical regions and hippocampus [1–4]. Previous studies have found that regional structures in the medial temporal lobe (MTL), hippocampus, posterior cingulate gyrus, and amygdala are especially vulnerable to Alzheimer’s disease [5–8]. Cortical atrophy extended progressively to the parietal and frontal cortex and sparing of sensorimotor areas until the late stages of AD [7, 9]. These structural deteriorations were associated with disease severity and the progression of clinical symptoms [10, 11]. These studies together suggested that Alzheimer’s disease differentially and specifically affects brain regions and imply a specific degenerative pattern [12].

Rather than focusing on specific brain regions, neuroimaging studies increasingly investigated large-scale brain networks, providing insights into the whole-brain organization. Diffusion tensor imaging and structural covariance network (SCN) analysis were commonly used methods to construct structural brain networks. These networks demonstrated distinct anatomical organization and morphological patterns during development, healthy aging, as well as in neurological and psychiatric diseases [13–17]. The SCNs revealed the coordinated patterns in brain morphology using inter-individual correlation of the structural measurements in regional volume, cortical thickness, and surface area [18–20]. The common approach to determining structural covariance was calculating the correlation of a structural feature (e.g., regional volume) between pairs of brain regions in a large sample of human individuals. Although the neurobiological basis of structural covariance remains unclear, it is probably the result of mutually trophic effects or experience-related plasticity and is also influenced by genetic and environmental factors [20–24].

SCNs exhibited specific trajectories across the lifespan [25]. Covariances in healthy aging reduced in cognitive and language networks but remained in sensorimotor regions [25, 26]. Organized networks with structural correlation were found to be targeted by neurodegenerative diseases and represent the syndrome-specific atrophy pattern [15, 27]. In the brain networks of AD, the default mode network regions, particularly in the posterior cingulate cortex, were widely reported to decrease structural connectivity, supporting one hypothesis of AD as a disconnection syndrome [28–31]. Meanwhile, increased structural covariances were observed in the salience and executive control networks of AD, which may reflect either overconnectivity or synchronized degeneration [18, 29, 32]. In the systems-level view, individuals with AD exhibited more localized, segregated, and less integrated whole-brain networks compared to healthy older adults [33–35]. While there was also a loss of small-world network characteristics in AD, previous studies have yielded inconsistent results regarding whether the networks became regular or random configurations [15, 36–38]. These analyses jointly suggested that mapping the structural patterns between brain regions could provide insights into the pathology of AD at the large-scale network level [18].

However, several challenges arise with the methods used for SCNs. Using population-level MRI data to construct SCNs might eliminate the inter-individual differences, limiting their clinical application in evaluating individual brain networks. Moreover, using Pearson’s correlation for a single structural feature, combined with the requirement for a large number of participants, might affect the stability of inter-regional structural patterns and ignore the distribution of intra-regional information [39, 40]. Recently, several innovative approaches have been developed to estimate morphological similarity and construct individual-level networks [39–46]. Using MRI data from an individual, one or multiple structural measurements are extracted to build an individual-based brain network. This network is based on the morphological similarities between brain regions, representing the intrinsic structural organization. To precisely depict the morphological distributions, Tijms et al. [41] proposed a method to divide the grey matter segmentation into 3 × 3 × 3 voxel cubes and correlate spatial patterns between two cubes. Kong et al. [42] further estimated regional morphological probability density and overcame the limitation associated with the rigid extraction of the cubes. In addition, recent studies combined multiple morphological indices (e.g., grey matter volume, surface area, curvature, and diffusion metrics) to construct morphometric patterns based on histological similarity accurately [39, 40, 47]. These studies together exhibited the potential of individual morphological networks in understanding cerebral organization and individual intrinsic interaction in brain structure and function. Since research on individual-based structural networks is still developing, few studies have focused on the micro-scale patterns and investigated the single-subject networks in individuals with AD [48, 49].

Given the limitations in previous studies, developing the approach to define the three-dimensional microscopic structures and more accurately estimate regional similarity may further elucidate the comprehensive morphological connection. Similar to the concept of regional homogeneity method, we identified the local structural patterns within the clusters of seven voxels [50]. We assumed that except for the grey matter intensity in a single voxel, the structural coordination within neighboring voxels reflects a specific morphological pattern and is disrupted in AD. To measure the inter-regional similarity by the probability distribution of structural patterns, we applied the information-based similarity (IBS) method [51]. The IBS method maps the sequence data into binary segments as specific patterns and compares the occurrences of different patterns between two sequences. By integrating the methods mentioned above, there is a potential to characterize local grey matter information to assess intrinsic architecture in brain regions and further investigate morphological changes in individual networks of AD. Our approach might provide new perspectives for future application in the precise personalized evaluation of individuals with AD. We aimed to develop a new approach for constructing individual morphological similarity based on structural patterns of grey matter voxels. Furthermore, we investigated the impacts of AD on morphological similarity between brain regions and regional structural organization. Our research on morphological similarity networks might provide comprehensive information on brain structural organization in AD.

## Materials and methods

### Participants

Data in the present study were sourced from the Alzheimer's Disease Neuroimaging Initiative (ADNI) database (https://adni.loni.usc.edu/). ADNI is a multicenter longitudinal study launched in 2003 and collects clinical, genetic, and imaging data from individuals across the AD clinical continuum. The clinical dataset includes demographics and cognitive assessment data of each participant. Besides, to establish imaging biomarkers in stages of the disease, MRI and PET data of participants are collected. Cognitively normal older adults (CN) without memory complaints were enrolled as control participants. The inclusion criteria were a Mini-Mental State Examination (MMSE) score of 24-30 points and a Clinical Dementia Rating (CDR) score of zero. Individuals with a subject memory complaint were diagnosed with AD requiring an MMSE score of 20-26 points, a CDR score of 0.5 or one, and a clinician’s diagnosis using NINCDS/ADRDA criteria for probable AD. All participants were assessed memory function by the Logical Memory II subscale from the Wechsler Memory Scale-Revised and scored according to their years of education. The stability of the allowed medications for 4 weeks was also checked. Participants with any significant neurologic disease other than AD were excluded. For up-to-date information, please see www.adni-info.org.

In this study, we incorporated the participants according to the following criteria. Participants had to 1) be diagnosed by criteria from ADNI and be categorized into the AD and CN groups; 2) be included in the screening or baseline visit; 3) have MRI images with a description of MPRAGE/MP-RAGE.

### MRI acquisition and preprocessing

MRI scans for ADNI-1 were performed on a 1.5T or 3T scanner. T1-weighted images were acquired using an MPRAGE sequence (repetition time = 2400 ms, echo time = 3 ms, flip angle = 8°, thickness = 1.2 mm, in-plane matrix size = 192 × 192, field of view = 240 × 240 mm^2^) [52]. MRI scans for ADNI-2 were performed at 3T with an MPRAGE protocol (repetition time = 2300 ms, echo time = 2.95 ms, flip angle = 9°, thickness = 1.2 mm, in-plane matrix size = 256 × 256, field of view = 260 × 260 mm^2^). For the participants with multiple visits, we selected the baseline scan for the following processing and analysis.

The preprocessing of structural MRI images was performed using the Data Processing Assistant for Resting-State fMRI (DPARSF) in the add-on toolbox for Data Processing & Analysis for Brain Imaging (DPABI) V4.3_200401 [53] and Statistical Parametric Mapping 12 (SPM12) running on MATLAB 2021a. After reorientation and skull stripping, images of all participants were normalized and transformed into MNI152 space. Finally, structural images were segmented into grey matter, white matter, and CSF of 2 mm × 2 mm × 2 mm voxels. Anatomical labeling of brain regions applied automated anatomical labeling (AAL) atlas [54].

### Information-based similarity analysis

#### Information-based similarity index

We conducted the information-based similarity method [51] to evaluate the similarity of structural patterns in brain regions between AD and CN groups. The method was developed to discover the hidden structure of sequence data and compare the similarity of two sequences. The information-based similarity index quantifies the distance (or dissimilarity) between two sequences using the occurrence proportion of defined elements. It has been applied to analyse symbolic sequences including heart rate time series [51], literary authorship disputes [55], and genetic sequences [56]. In these studies, the “words” were elements defined in a given length, representing a unique pattern in the sequence (e.g., fluctuations in time series and n-tuple nucleotides in genetic sequences). These words are then ranked according to their occurrence probabilities in the sequence in descending order. The first rank word corresponds to the most common pattern in the symbolic sequence. Using the rank order of words, the weighted distance, denoted as D and ranging from zero to one, defined the dissimilarity of two sequences, S_1_ and S_2_.

1 $$D({S}_{1},{S}_{2})=\frac{1}{{N}_{12}}\sum_{k=1}^{{N}_{12}}|{R}_{1}\left({w}_{k}\right)-{R}_{2}\left({w}_{k}\right)|F\left({w}_{k}\right)$$

The weighting function F(w_k_) derived from Shannon's entropy is as follows, where Z indicates the normalization factor.

2 $$F\left({w}_{k}\right)=\frac{1}{Z}\left[-{p}_{1}\left({w}_{k}\right){\text{log}}\left({p}_{1}\left({w}_{k}\right)\right){-p}_{2}\left({w}_{k}\right){\text{log}}\left({p}_{2}\left({w}_{k}\right)\right)\right]$$

3 $$\sum_{k=1}^{{N}_{12}}F\left({w}_{k}\right)=1$$

Here R_1_(w_k_) and p_1_(w_k_) denote the rank and probability of a given word, w_k_, in the sequence S_1_. R_2_(w_k_) and p_2_(w_k_) represent the same in sequence S_2_. N_12_ is the total number of shared words in sequence S_1_ and S_2_.

#### Structural pattern index

To capture the structural pattern in micro-scale (i.e., words in the IBS method), we defined the structural pattern indices by relationships of grey matter intensity in adjacent voxels relative to a given voxel. For a voxel x, its grey matter intensity was compared to its six adjacent voxels. If the grey matter intensity in voxel x was greater than or equal to its neighbor voxel n (v1, v2, …, v6), the symbol Sn (S1, S2, …, S6) was marked with a one; otherwise, it was marked with a zero.

4 $$Sn=\left\{\begin{array}{c} 0, vx<vn\\ 1, vx\ge vn\end{array}\right.$$

**Fig. 1:**
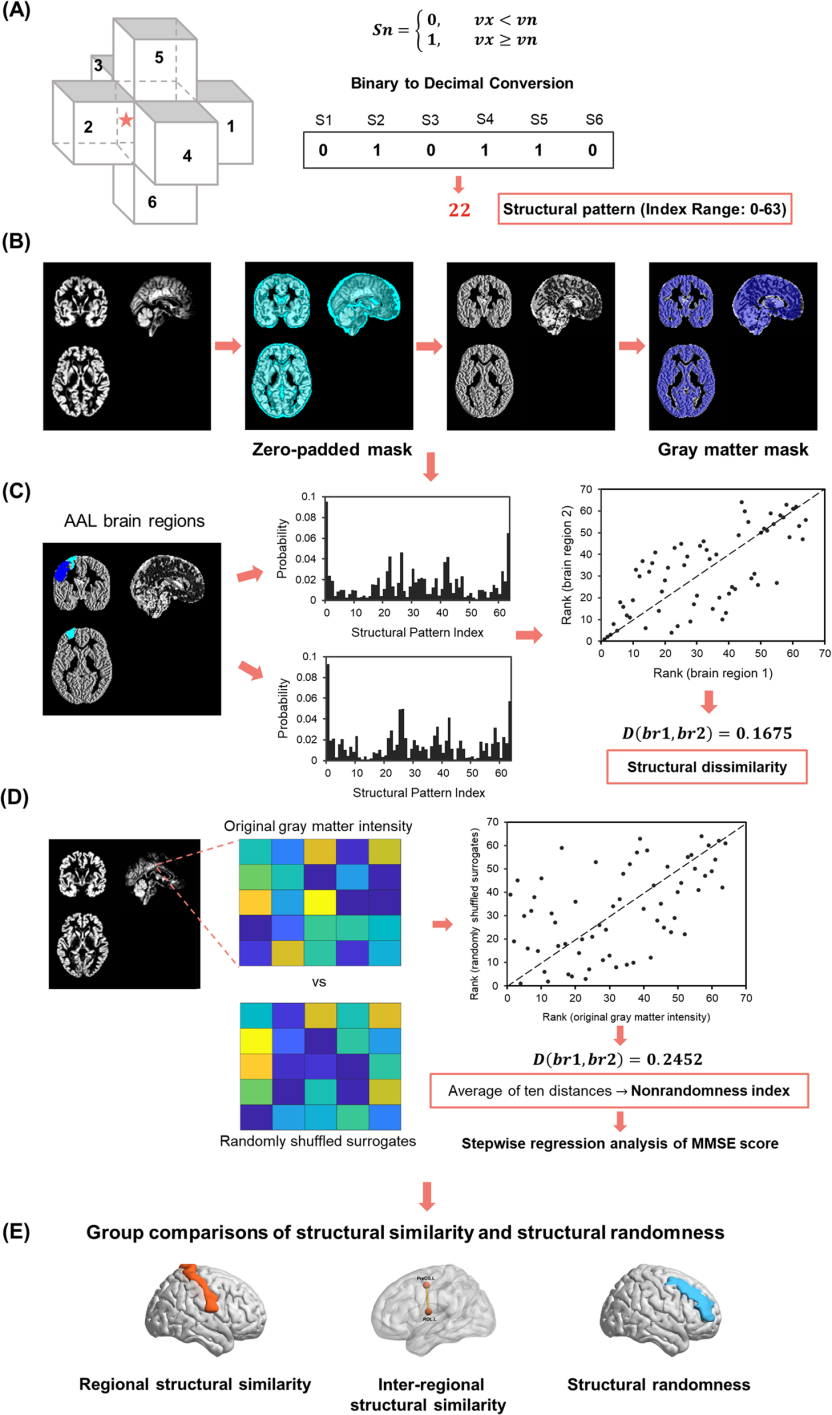
Flowchart. **A** Grey matter relationships with neighboring six voxels identified structural pattern indices. The binary symbol sequence, which represented the combination of intensity relationships, was then converted to a decimal pattern index. **B** Preprocessed grey matter maps were mapped using a zero-padded grey matter mask. This step ensured the retention of voxels and their neighbors for morphological similarity analysis. Subsequently, these were mapped with the grey matter mask. **C** We measured the IBS distances as dissimilarity between two AAL brain regions based on the probabilities and rank orders of structural patterns. **D** The IBS distance between the original and spatial shuffled grey matter intensities assessed the structural randomness. The stepwise regression was used to examine the relationship of the MMSE score with structural randomness. **E** We applied the one-sample t-test to investigate the regional structural similarity and the independent t-tests to explore group differences in inter-regional structural similarity and structural randomness. AAL: automated anatomical labeling; IBS: information-based similarity; MMSE: Mini-Mental State Examination

We mapped the six symbols to a binary sequence, following the order: right, left, anterior, posterior, superior, and inferior voxels. The binary sequence, representing intensity relationships, was then converted into a decimal number (see more details in Fig. 1A). These decimal numbers, identified as structural pattern indices, were integers between zero and 63 (i.e., 2^6^ = 64 combinations), with each index corresponding to a unique spatial pattern.

To ensure grey matter voxels and their neighbors were included in the analysis and to preserve the boundary information, the preprocessed structural MRI data of all participants were mapped with a zero-padded grey matter mask. This study only included grey matter voxels with six intact neighboring voxels. Next, we filled the structural pattern indices into the corresponding voxels to generate a map for each participant. The resulting images were mapped with the standard grey matter mask again and applied for morphological similarity analysis (Fig. 1B).

#### Structural similarity

In this study, we performed a word rank frequency analysis on 64 structural pattern indices in a specific brain area. We calculated the occurrence probability of each index and sorted it in descending order. Subsequently, the IBS distance between two brain areas was measured based on their respective index rank orders, allowing us to assess inter-regional structural similarity (Fig. 1C). The small IBS distances between brain regions are expressed as structural similarity.

We conducted two sections of examination to explore the alterations in structural similarity in AD. First, we computed the index rank for each brain region based on the group average probability of each index. The similarity between a specific brain region of the two groups was then defined as the regional structural similarity. Brain areas with small regional structural similarity to the CN group represented the affected regions in AD. Second, we calculated the inter-regional similarities between pairs of brain regions, resulting in 4005 (C(90,2) = 4005) structural similarities for each participant. The inter-regional similarities were examined to investigate whether the morphological associations varied with AD progression.

#### Structural randomness

To quantify the organizational characteristics of structural patterns in a brain region, we calculated the nonrandomness index derived from the IBS method [51]. The nonrandomness index is the average IBS distance between the original signal and its randomized surrogates. A high nonrandomness index means that the original signal is not similar to the randomized one, suggesting that it is a more regular organization. In contrast, a low nonrandomness index indicates a more random configuration. The nonrandomness index was applied to quantify underlying dynamics features of heart rate time series and effectively discriminate the healthy subjects and subjects with congestive heart failure [51].

In addition to structural similarity reflecting structural coordination between groups and brain regions, we explored structural randomness to find the underlying organization of regional structure. The IBS distance between the structural pattern of the original and voxel-shuffled grey matter density map evaluated the degree of structural nonrandomness. For every AAL brain region, we generated a regional zero-padded mask to extract neighboring voxels. Next, we created ten randomly shuffled surrogates, remaining with the grey matter intensity information but disrupting the spatial distribution. Each surrogate was denoted with voxel-wise structural pattern indices and compared the IBS distance to the structural pattern map derived from the raw grey matter intensity. Finally, the average of ten distances was computed as the nonrandomness index for each brain region (Fig. 1D). The low nonrandomness index of a brain region is denoted as structural randomness.

### Statistical analysis

For the demographic characteristics, we used the independent t-test and chi-square test for the statistical analysis of continuous and categorical demographic variables respectively. The *P* value was set at 0.05.

We performed a one-sample t-test to investigate the regional structural similarities. The significantly large IBS distances were examined, setting the significance level at *P* < 0.05 (right-tailed). The comparison was used to identify the brain regions dissimilar in structural pattern between the CN and AD groups. After calculating 4005 inter-regional structural similarities for each participant, we performed the independent t-test for each inter-regional structural similarity to examine group differences. To correct for multiple comparisons, we used the Manhattan plots to investigate the threshold and determined the significance at a *P* < 10^-12^ (Supplementary Fig. 1 and Supplementary Fig. 2).

We performed the independent t-test on group comparisons in structural randomness. The Bonferroni correction was applied for multiple comparisons with *P* < 5.56 × 10^-4^. Moreover, we evaluated the relationship between the regional structural randomness and the cognitive performance of individuals with AD. In the regression models, the dependent variable was the MMSE score of individuals with AD and the nonrandomness indices of brain regions were the independent variables. We conducted the linear regression to identify potential brain regions with the significance level of *P* < 0.1. After normalizing the nonrandomness indices for these brain regions, we used the stepwise regression analysis using the stepwiselm function in MATLAB to identify brain regions with the significant association. We controlled for age, sex, and education using the generalized linear regression model. The significance level was set at 0.05. Finally, all results were visualized by the BrainNet Viewer (http://www.nitrc.org/projects/bnv/) [57].

## Results

### Demographic characteristics

We recruited T1-weighted images and cognitive assessment data from 342 cognitively normal participants and 276 individuals with AD from the ANDI-1 and ADNI-2 studies. There were no age and sex differences between the CN group (mean age = 75.41 years, 178 females) and the AD group (mean age = 75.08 years, 130 females). Besides, significant group differences were found in the year of education and MMSE score (CN group: mean education = 16.28 years and mean MMSE score = 29.12; AD group: mean education = 15.19 years and mean MMSE score = 23.20). Individuals with AD had a mean disease duration of 3.91 years. The demographics of the two groups are reported in Table 1.

**Table 1:** Demographics

	**CN (*n* = 342)**	**AD (*n* = 276)**	***P* Value**
Age, years	75.41 ± 6.11	75.08 ± 7.74	0.57^a^
Sex, female, n (%)	178 (52%)	130 (47%)	0.20^b^
Education, years	16.28 ± 2.68	15.19 ± 3.02	<0.0001^a^
Duration of AD, years	-	3.91 ± 2.89	-
MMSE	29.12 ± 1.08	23.20 ± 2.09	<0.0001^a^

Data are presented as mean ± SD unless otherwise stated

*CN* Cognitively normal older adults, *AD* Alzheimer's disease

^a^independent t-test, ^b^chi-square test

### Group differences in regional structural similarity

**Fig. 2:**
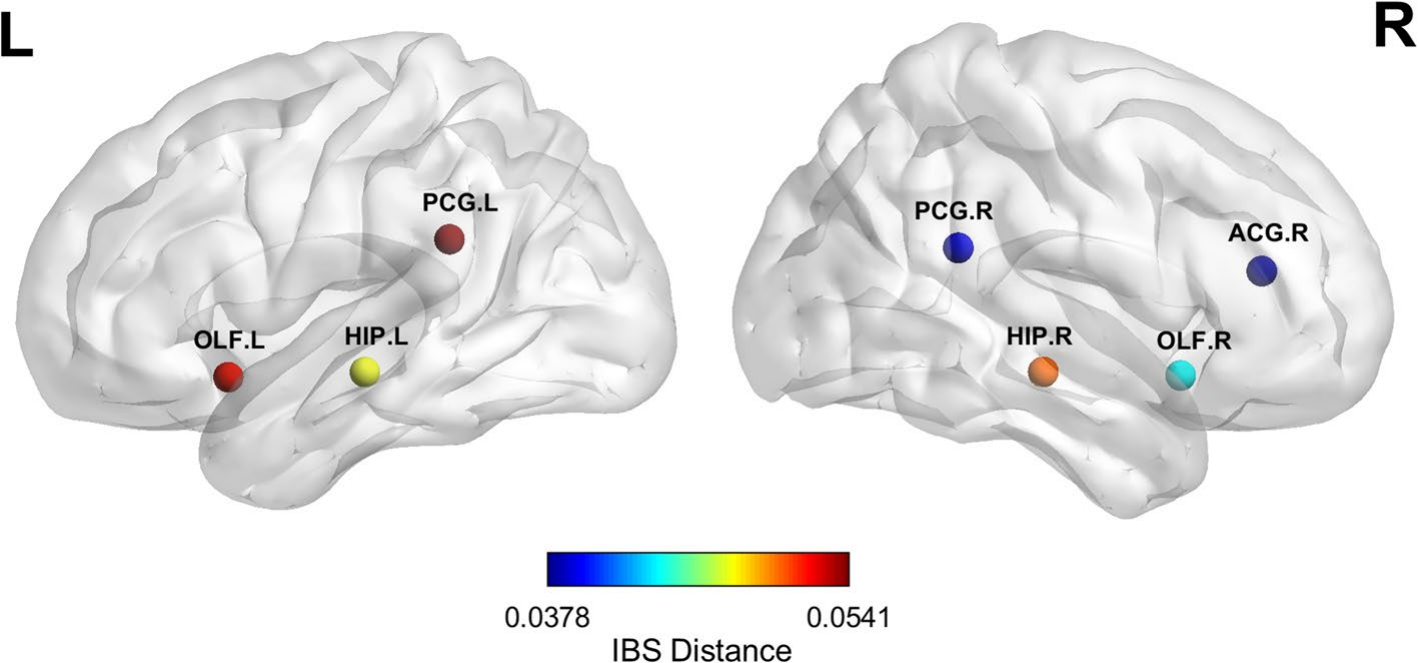
Brain regions with significant difference in regional structural similarity. We first generated the group average probability of structural pattern indices. Next, we calculated the distances (dissimilarities) for the same region between the CN and AD groups. The nodes represent the brain regions with significant dissimilarity, and the colors illustrate the IBS distances. AD: Alzheimer’s disease; CN: cognitively normal older adults; IBS: information-based similarity; ACG: anterior cingulate and paracingulate gyri; HIP: hippocampus; OLF: olfactory cortex; PCG: posterior cingulate gyrus

For each brain region, we compared the regional structural similarity between the AD and CN groups, reflecting alterations in the combination of structural patterns in individuals with AD. Significant group differences in regional structural similarity were found in the bilateral posterior cingulate gyrus, olfactory cortex, hippocampus, and right anterior cingulate and paracingulate gyri (Fig. 2). Supplementary Table 1 provides the full group dissimilarities of brain regions.

### Group differences in inter-regional structural similarity

**Fig. 3:**
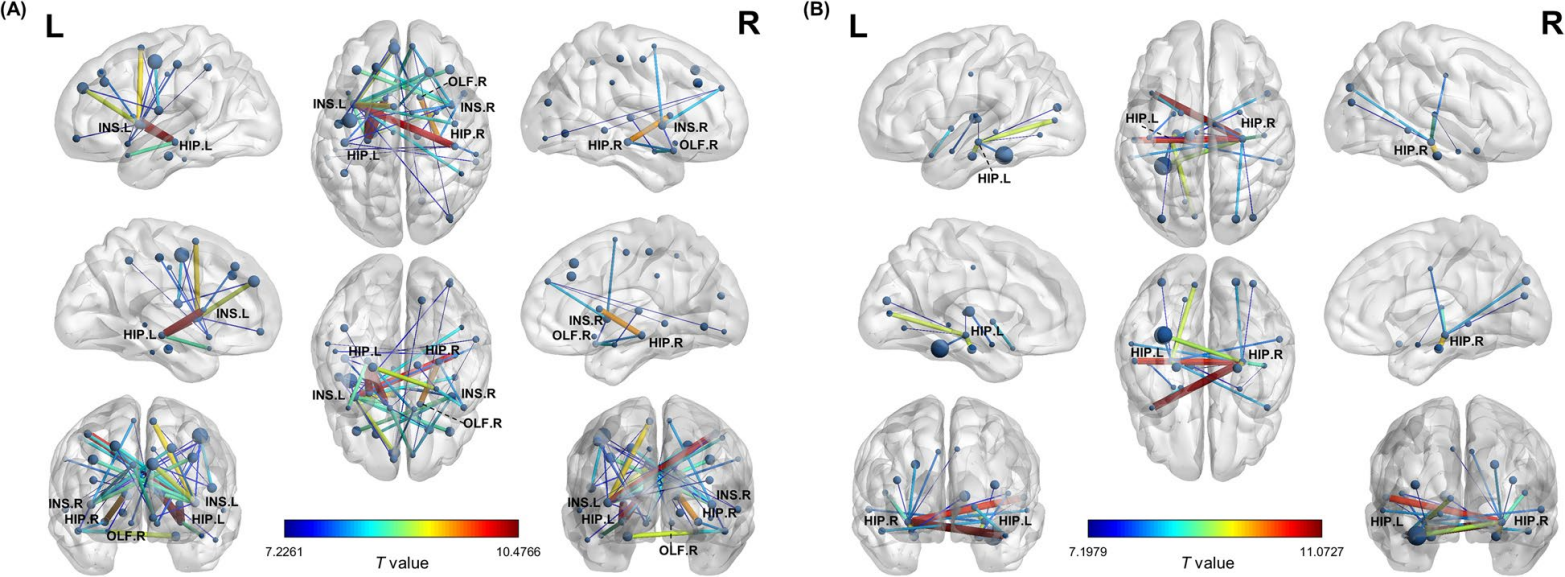
Altered inter-regional structural similarities in AD. We calculated the inter-regional distances to evaluate the individual morphological similarities in all participants. **A** The nodes represent brain regions where individuals with AD showed significantly decreased inter-regional structural similarity. **B** The nodes represent the brain regions where inter-regional structural similarity significantly increased in individuals with AD. The edges illustrate pairs of altered similarities, with colors representing the t values from comparisons between AD and CN groups. AD: Alzheimer’s disease; CN: cognitively normal older adults; HIP: hippocampus; INS: insula; OLF: olfactory cortex

We calculated inter-regional similarity to evaluate the individual morphological brain network in all participants. Compared to the CN group, the left and right insula of the AD group showed decreased similarity to 14 and seven other brain regions, respectively. Most of these regions were located in the frontal and parietal lobes, including the postcentral gyrus, supplementary motor area, middle frontal gyrus, and medial frontal gyrus. The right olfactory cortex of individuals with AD also had decreased similarity to six brain regions, notably the superior frontal gyrus, postcentral gyrus, and supplementary motor area. In the temporal and subcortical regions, the bilateral hippocampus showed decreased similarity to the ipsilateral caudate nucleus and temporal pole (Fig. 3A).

Meanwhile, the left and right hippocampus of individuals with AD revealed increased similarity to eight and 11 other brain regions. These regions were found in the temporal, subcortical, and occipital areas, particularly in the contralateral temporal pole, middle temporal gyrus, ipsilateral parahippocampal gyrus, left superior temporal gyrus, and left fusiform gyrus (Fig. 3B). Supplementary Table 2 presents the full details of group differences in inter-regional structural similarity.

### Altered structural randomness in AD

**Fig. 4:**
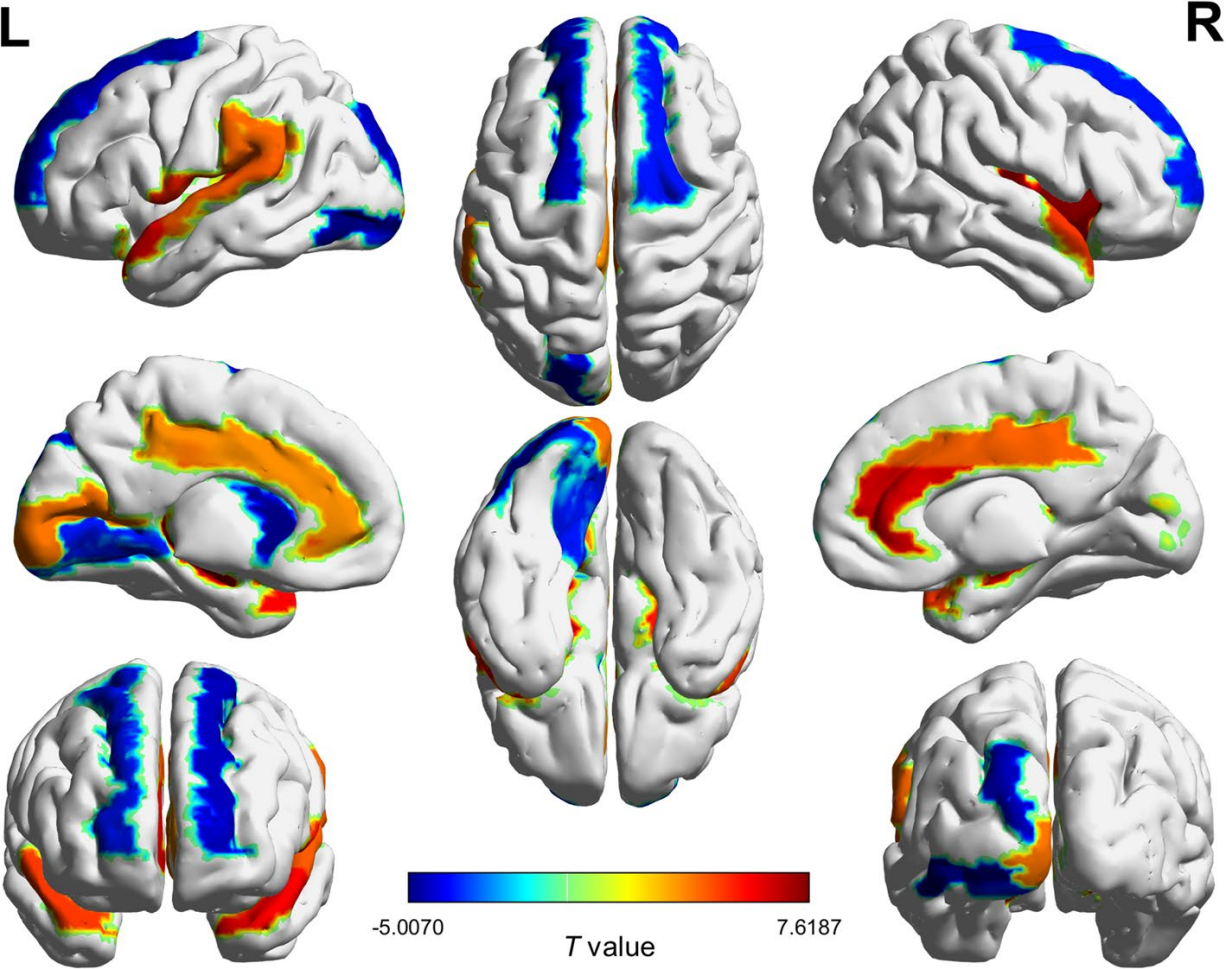
Group differences in structural randomness of brain regions. We calculated the nonrandomness index for each brain region and investigated the group differences. The colors represent the t values from group comparisons between AD and CN groups. AD: Alzheimer’s disease; CN: cognitively normal older adults

We calculated the nonrandomness index of each brain region to investigate the differences in structural pattern organization between the two groups. Compared to the CN group, individuals with AD significantly decreased in structural randomness in the temporal and subcortical regions. Specifically, this decrease was evident in the right insula, bilateral hippocampus, anterior cingulate and paracingulate gyri, and temporal pole. Meanwhile, significant increases in structural randomness were found in the occipital and frontal regions, notably in the left inferior occipital gyrus, left caudate nucleus, and bilateral superior frontal gyrus. Fig. 4 illustrates the brain regions with alternation in structural randomness (see Supplementary Table 3 for full comparisons).

### Association of structural randomness with cognitive performance in AD

**Fig. 5:**
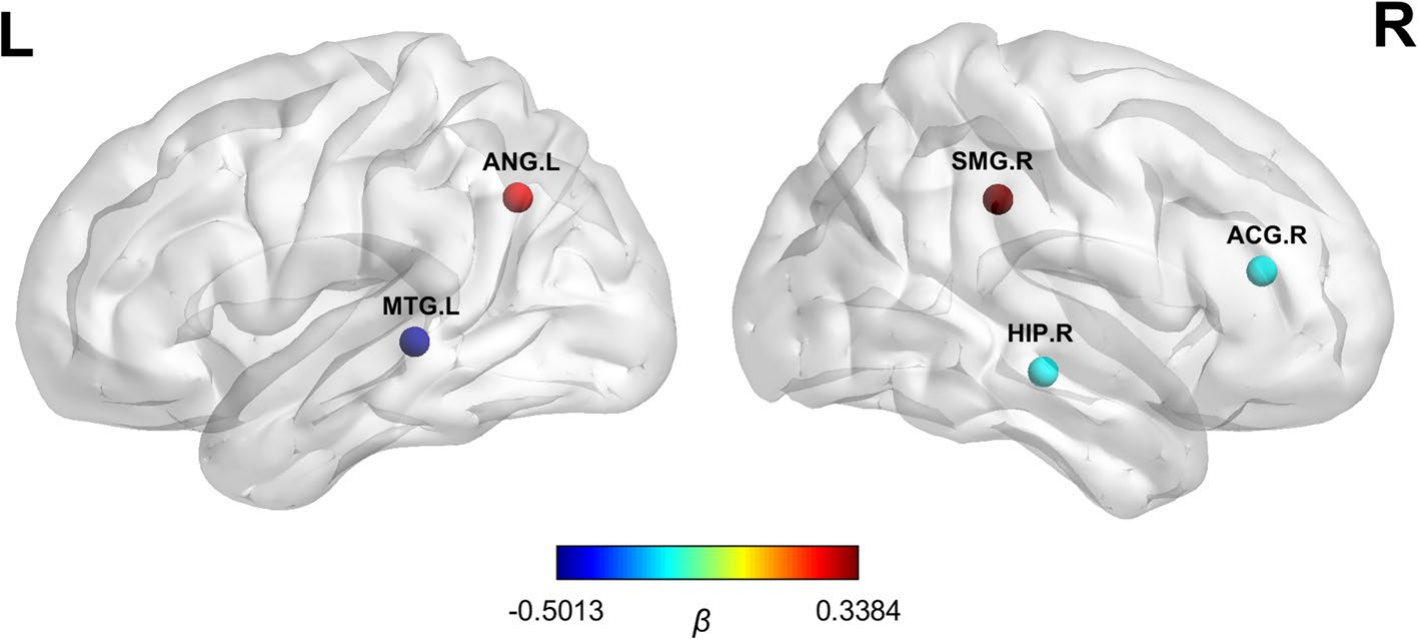
Brain regions with structural randomness associated with cognitive performance in AD. The nodes represent the brain regions where the nonrandomness indices correlated with the cognitive performance in individuals with AD. The colors illustrate the beta coefficients derived from the stepwise regression analysis. AD: Alzheimer’s disease; ACG: anterior cingulate and paracingulate gyri; ANG: angular gyrus; HIP: hippocampus; MTG: middle temporal gyrus; SMG: supramarginal gyrus.

In individuals with AD, we investigated the relationship between the structural randomness feature and cognitive performance using a regression analysis. The result showed that the MMSE score was associated with the structural randomness in the right supramarginal gyrus (*β* = 0.34, SE = 0.09, *P* < 0.001), left angular gyrus (*β* = 0.26, SE = 0.09, *P* = 0.003), right anterior cingulate and paracingulate gyri (*β* = -0.20, SE = 0.08, *P* = 0.009), right hippocampus (*β* = -0.20, SE = 0.08, *P* = 0.01), and left middle temporal gyrus (*β* = -0.50, SE = 0.10, *P* < 0.001) (see more details in Fig. 5 , Supplementary Fig. 3, and Supplementary Table 4).

## Discussion

In this study, we developed a novel method to identify micro-structural patterns, enabling the estimation of morphological similarity and structural randomness based on regional pattern configuration. The present study has four main findings. First, large-scale alterations in morphological similarity were found in the AD group. Compared to the CN group, the AD group exhibited decreased inter-regional structural similarities between the insula and frontal and parietal regions. Second, the hippocampus increased inter-regional structural similarities with the temporal, subcortical, and occipital areas in individuals with AD. Third, individuals with AD had a more regular morphological organization in the temporal and subcortical regions, while the occipital and frontal areas tended to have a more randomized arrangement of structural patterns. Finally, our stepwise regression analysis showed that the morphological randomness in five brain regions of individuals with AD was associated with overall cognitive performance.

### Methodology of morphological similarity network

To the best of our knowledge, this is the first study using the local structural features—based on the grey matter intensity relationship between neighboring voxels—to construct structural patterns and investigate the individual inter-regional similarity. Grey matter volume and cortical thickness were initially used in past studies of structural networks to measure regional morphology. Recent studies extended to include surface-based indices, geometric measures, and diffusion metrics through multimodal MRI data to capture microstructure and complement different morphological information [39, 40, 43, 47]. In addition to regional structure, Tijms et al. [41] extracted the grey matter density in the cubes of 27 voxels to preserve the three-dimensional structure and spatial information. This approach reflected the local features and speculated the possible mechanism of intracortical similarities as axon tension theory [58]. The theory proposed that connected cortical areas are pulled by a mechanical force and become thinner or thicker. Hilgetag & Barbas [59, 60] also proved that axon-connectivity had an influence on morphology by the tracer studies in the primate brains. Moreover, Seidlitz et al. [40] examined the consistency between the distribution of morphological similarity and cortical cytoarchitecture using MRI and diffusion-weighted imaging (DWI) data. They found a strong association between morphometric similarity and both cytoarchitectonic and genomic measures of histological similarity, further linking this association to axonal connectivity [61, 62]. These findings supported that morphological similarity based on MRI measurements could serve as an approximate marker for axonal connectivity with histological validity. Seidlitz et al. [40] also compared structural networks constructed only by structural covariance or diffusion tractography. The performance in cytoarchitectonic alignment was found to be relatively weaker in DWI networks. This difference might arise from challenges associated with reconstructing long-distance and interhemispheric connections [63]. Our method focused on local patterns of grey matter and annotated within the anatomical regions, which might preserve more accurate and intact morphological information. Because we identified the relationship of adjacent voxels in the determined order, future studies could potentially infer morphological changes from the structural pattern indices. The advancement of ultra-high field MRI has enabled the imaging of micro-anatomic structures, including cortical layers. Kenkhuis et al. used 7T T2*-weighted MRI to observe disturbances in cortical lamination of MTL in individuals with AD [64]. Mapping the distribution of morphological similarity and layered cortical structure may complement information on specific patterns of atrophy. Finally, further research is warranted on extracting information from the voxels near brain regional boundaries and constructing reproducible similarity networks with a biological basis.

### Morphological pattern in cognitively normal older adults

We first evaluated the morphological similarity in cognitively normal older adults to demonstrate the inter-regional properties constructed by our method. The detailed results of the CN group can be found in the supplementary information. In the CN group, we observed that several regions, including bilateral precentral gyrus, middle frontal gyrus, right postcentral gyrus, left superior parietal gyrus, right supramarginal gyrus, and left precuneus showed structural patterns similar to many other brain regions. These regions were located at the sensorimotor and association cortex. Montembeault et al. [65] found that the sensorimotor cortex remained stable in structural covariance during normal aging. Meanwhile, we noted the regions with structural similarity also shared spatial proximity. Our results were consistent with previous findings that the strength of structural similarity was inversely related to the physical distance between regions [18, 66]. Oppositely, the bilateral globus pallidus (GP) were dissimilar in structural pattern to other brain areas. Past studies have subdivided the GP into an internal and an external segment and found topographically and functionally segregated clusters and pathways in the GP [67, 68]. These findings jointly suggested that cortical regions involved in the same function were similar in structural pattern, while subcortical areas with subdivisions had their unique structural arrangement. Furthermore, our method may reflect the property of structural similarity between brain regions based on cortical function and topological arrangement.

Moreover, we found lower structural randomness in the bilateral amygdala, left temporal pole, and left thalamus, indicating a more regular configuration of the structural patterns in these regions. The amygdala, comprises a collection of nuclei, is regarded as the integrative core of emotion. Previous findings have highlighted the amygdala was observed to tend to retain structural integrity during normal aging [69, 70]. Concurrently, the temporal pole regions and thalamus, characterized by their complex structures and involved in high-order cognitive functions and relaying information, also showed relative preservation in the age-related grey matter alteration [71–73]. Although previous studies have yielded inconsistent results on structural changes in these regions, we speculated that brain regions with complex structure and function might have unique and stable structural organization, as reflected by the structural randomness.

### Regional structural similarity altered in individuals with AD

In the AD group, we found significant differences in regional structural similarity in the bilateral posterior cingulate gyrus, olfactory cortex, hippocampus, and right anterior cingulate and paracingulate gyri compared to the CN group. These brain areas were detected as structural atrophy and vulnerable to AD [7, 74, 75]. Past studies found the posterior cingulate cortex and hippocampus as core regions in the structural network in the early stage of AD. The degenerations in these regions were observed earlier and could predict atrophy in other brain regions, correlating with the progression of AD [76, 77]. Our results further revealed that vulnerable brain regions in AD might disrupt the configuration of structural patterns rather than homogeneous atrophy in all regions.

### Altered inter-regional structural similarity in individuals with AD

Compared to the CN group, we observed a decrease in inter-regional structural similarity between bilateral insula and both frontal and parietal regions in individuals with AD. The insula is the crucial hub of the human brain networks, integrating information from different functional systems that support sensory, emotional, motivational, and cognitive processing [78, 79]. Studies in nonhuman primates have described widespread structural connections of the insula with frontal, parietal, temporal, and subcortical regions [79, 80]. In human studies, Ghaziri et al. [81] performed structural connectivities between the insula and several regions, including the orbitofrontal cortex, the supplementary motor area, the primary motor, and the somatosensory cortices. Meanwhile, the insular cortex was found to be vulnerable to AD pathology and altered functional connectivity in individuals with mild cognitive impairment (MCI) and AD [82–84]. Xie et al. [84] demonstrated that both anterior and posterior insula networks exhibited decreased positive connectivity in the dorsolateral prefrontal cortex and temporal pole in individuals with amnestic MCI. These brain regions were aligned with our findings of decreased inter-regional structural similarities to the insula.

Additionally, we noted reduced inter-regional similarities between the bilateral hippocampus and the ipsilateral caudate nucleus. The hippocampus plays a crucial role in learning and memory, and its impairment is widely associated with episodic memory deficits in the early stage of AD [85]. Furthermore, the hippocampus is a central structure in various memory systems and interacts with various regions across different brain networks [86, 87]. Several studies have indicated that the ipsilateral interaction between the hippocampus and the caudate nucleus involved navigation and map-based spatial memory [86, 88, 89]. The topographical disorientation in AD was characterized as clinical significance and might serve as a potential biomarker for detecting AD in the preclinical stages [90, 91]. Taken together, our findings supported the disconnection hypothesis of AD and implied the probable relationship between dysfunction and micro-scale morphological patterns.

In the meantime, we observed increased inter-regional similarities between the hippocampus and the temporal, subcortical, and occipital regions in individuals with AD. These regions included the temporal pole, middle temporal gyrus, parahippocampal gyrus, left superior temporal gyrus, and left fusiform gyrus. While many studies have reported disrupted inter-regional connectivity in individuals with AD, a few have observed increased connectivity. In particular, studies of structural and functional networks in AD have found increased connections in salience networks [32, 92]. Dautricourt et al. [93] found increased functional connectivity between the anterior hippocampus and perirhinal cortex seeds and left anterior medial temporal regions in individuals with AD compared to controls. The increased structural covariance may represent the hyperconnectivity or synchronized deterioration between brain regions [18]. To interpret the clinical implications of the structural similarity and elucidate the underlying mechanism, more research is warranted, especially including participants with severe AD and incorporating functional data.

### Structural randomness and its relationship to cognitive performance

Individuals with AD had a significantly lower level of structural randomness in the temporal and subcortical areas, including the right insula, bilateral hippocampus, anterior cingulate and paracingulate gyri, and temporal pole. Meanwhile, the occipital and frontal regions showed increases in randomness, notably in the left inferior occipital gyrus, left caudate nucleus, and bilateral superior frontal gyrus. Importantly, we focused on the intra-regional morphological organization rather than properties between regions. Bonthius et al. [82] found that the severity of insular pathology was affected by different cytoarchitectonic arrangements in individuals with AD. Our results showed that brain regions vulnerable to the early stage of AD tended to have a more regular pattern arrangement. The occipital and frontal regions affected in the late stage of AD reveal a more random configuration of structural patterns. Together, these findings suggested that the regular morphological configuration may be vulnerable to pathology and associated with regional atrophy. Further research is needed to clarify the biological mechanism and reliability.

Furthermore, we found an association between the MMSE score in individuals with AD and the morphological randomness in the right supramarginal gyrus, left angular gyrus, right anterior cingulate and paracingulate gyri, right hippocampus, and left middle temporal gyrus. The angular gyrus, a part of the parietal association cortex, is considered the major connecting hub of complex language functions [94]. Anatomical tracer studies have illustrated the structural connection between the angular gyrus and posterior supramarginal gyrus, middle temporal gyrus, and hippocampus [95–97]. These brain regions were consistent with our results and functionally involved in semantic processing and episodic memory retrieval [94, 98]. Individuals with AD exhibited measurable semantic and memorial deficits in the early disease stage [85, 99, 100]. Taken together, our results suggested that the structural organization of specific brain regions might have a relationship with cognitive function, potentially reflecting the factors affecting cognitive performance.

### Limitations

The present study still comes with some limitations. First, we used a cross-sectional design to explore morphological similarity in AD. It is essential to confirm the progression of structural patterns in one individual using longitudinal data in future studies. Second, MRI data from ADNI-1 were acquired at 1.5T and 3T. Although the data distributions of field strengths match in the two groups, we could not completely rule out the probable effect. Furthermore, we did not apply graph-based network analysis but focused on inter-regional similarity. Finally, we only used MMSE to assess the cognitive performance of participants. MMSE was often observed with the ceiling effect and might not detect early cognitive impairment reliably [101]. Incorporating a broader range of cognitive assessments could help clarify the relationship between cognitive functions and morphological similarity.

## Conclusions

In conclusion, the present study developed a novel method to construct individual morphological similarity networks based on the grey matter intensity relationship between neighboring voxels. With this approach, we found individuals with AD had decreased inter-regional structural similarity between the insula and frontal regions, while the hippocampus revealed increased inter-regional structural similarity to temporal and subcortical regions. In addition, we observed decreased structural randomness in the temporal and subcortical regions while increased structural randomness in the occipital and frontal regions. Notably, the MMSE score in individuals with AD was associated with structural randomness in five brain regions. Our morphological similarity network approach could provide valuable insight into the whole-brain structural organization of AD.

### Supplementary Information

**Supplementary Material 1.** 

## Data Availability

Data used in these analyses are available from the ADNI studies through the Image and Data Archive website (https://ida.loni.usc.edu/).

## Abbreviations

AAL: Automated anatomical labeling
AD: Alzheimer’s disease
CN: Cognitively normal older adults
IBS: Information-based similarity
MMSE: Mini-Mental State Examination
SCN: Structural covariance network
