# Supplementary material for: Exploring morphological similarity and randomness in Alzheimer’s disease using adjacent grey matter voxel-based structural analysis
Source: Alzheimers Res Ther. 2024 Apr 23;16:88. doi: 10.1186/s13195-024-01448-1 (PMC11036786; doi:10.1186/s13195-024-01448-1)
Supplement: Supplementary file 1 — Supplementary Material 1. [file 13195_2024_1448_MOESM1_ESM.pdf]

Exploring morphological similarity and randomness in Alzheimer's disease using adjacent grey matter voxel-based structural analysis

– SUPPLEMENTARY MATERIAL –

Ting-Yu Chen, Jun-Ding Zhu, Shih-Jen Tsai, Albert C. Yang*

Methods and Results of the Cognitively Normal (CN) Group

Statistical analysis

We performed a one-sample t-test to examine the following data. First, we explored the trend in inter-regional structural similarity in the CN group, setting the significance level at $P < 0.05$ (two-tailed uncorrected). Second, we examined the mean structural randomness of brain regions in the CN group, with a significance threshold of $P < 0.05$ (two-tailed).

Structural similarity and randomness in the CN group

We estimated the inter-regional structural similarity in the CN group to investigate the similarity constructed by voxel-based patterns. Brain regions with a significantly small distance (or similar) to more than four other regions included bilateral precentral gyrus, middle frontal gyrus, right postcentral gyrus, left superior parietal gyrus, right supramarginal gyrus, and left precuneus. Supplementary Fig. 4 presents brain regions similar in structural pattern to the right precentral gyrus, which also had spatial proximity. Meanwhile, the left and right globus pallidus (GP) presented significantly large distances (or dissimilar) to 56 and 52 other brain areas, respectively. Structural similarities between 90 brain regions in the CN group can be found in Supplementary Table 5.

To examine the property of structural pattern organization, we computed the average nonrandomness index of each brain region in the CN group. We found a significantly lower mean structural randomness in the bilateral amygdala, indicating a more regular arrangement of the structural patterns. The higher structural randomness was also observed in the temporal and subcortical regions, particularly in the left temporal pole and thalamus. Additionally, the right calcarine fissure and surrounding cortex revealed a significantly higher mean structural randomness, suggesting a more random morphological organization. We also observed the structural randomness in the parietal and occipital regions. Supplementary Fig. 5 and Supplementary Table 6 demonstrate the whole brain distribution of average structural randomness in the CN group.

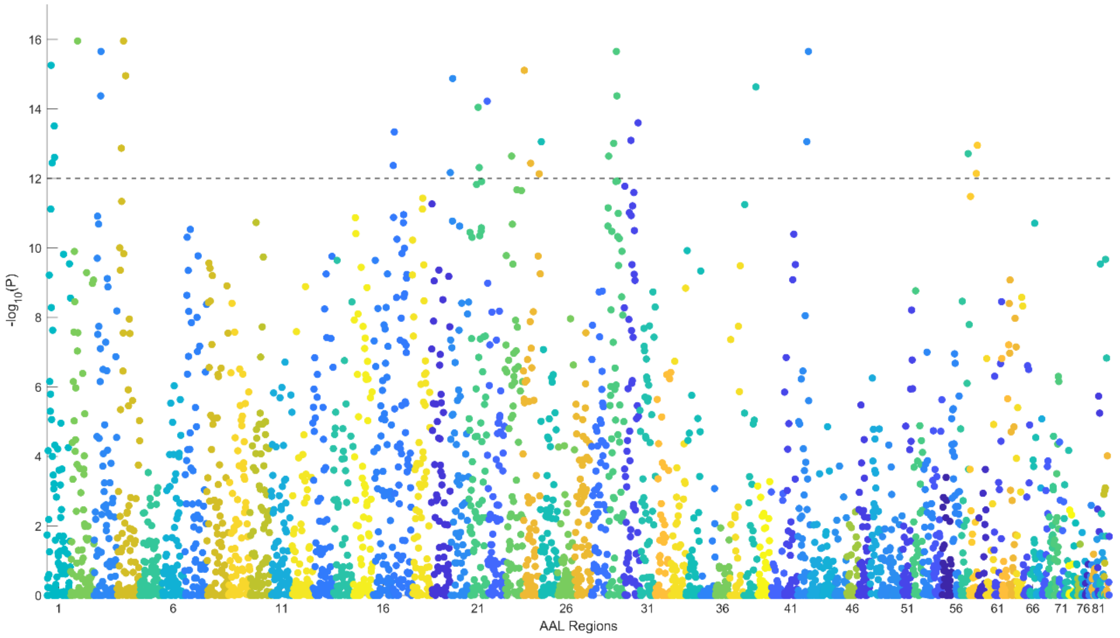

Supplementary Figure 1 Manhattan plot of increased structural dissimilarities in Alzheimer's disease. The Manhattan plot illustrates -log$_{10}$ of the *p* value in group comparisons of inter-regional dissimilarities in which individuals with AD were greater than cognitively normal older adults. These comparisons represent the decreased similarity in individuals with AD. The dots represent 4005 pairs of dissimilarity (C_2^{90} = 4005). The colors of the dots present the reference AAL region of the comparisons. We determined the significance threshold at 10^{-12}. AAL: automated anatomical labeling.

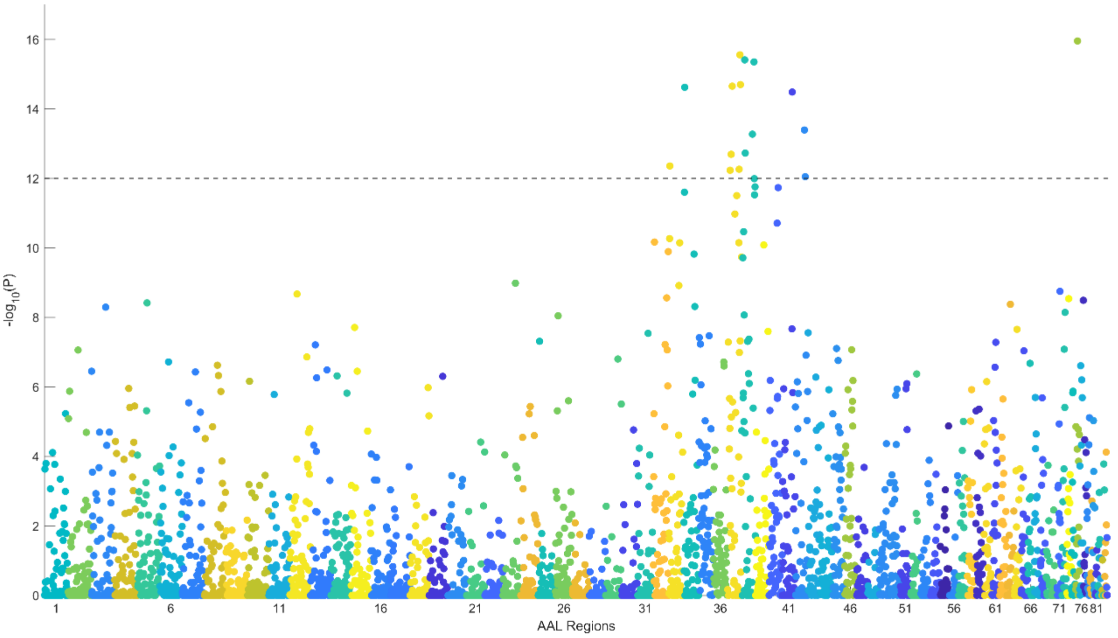

Supplementary Figure 2 Manhattan plot of decreased structural dissimilarities in Alzheimer's disease. The Manhattan plot illustrates -log10 of the p value in group comparisons of inter-regional dissimilarities in which individuals with AD are less than cognitively normal older adults. These comparisons represent the increased similarity in individuals with AD. The dots represent 4005 pairs of dissimilarity (C_2^{90} = 4005). The colors of the dots present the reference AAL region of the comparisons. We determined the significance threshold at 10^{-12}. AAL: automated anatomical labeling.

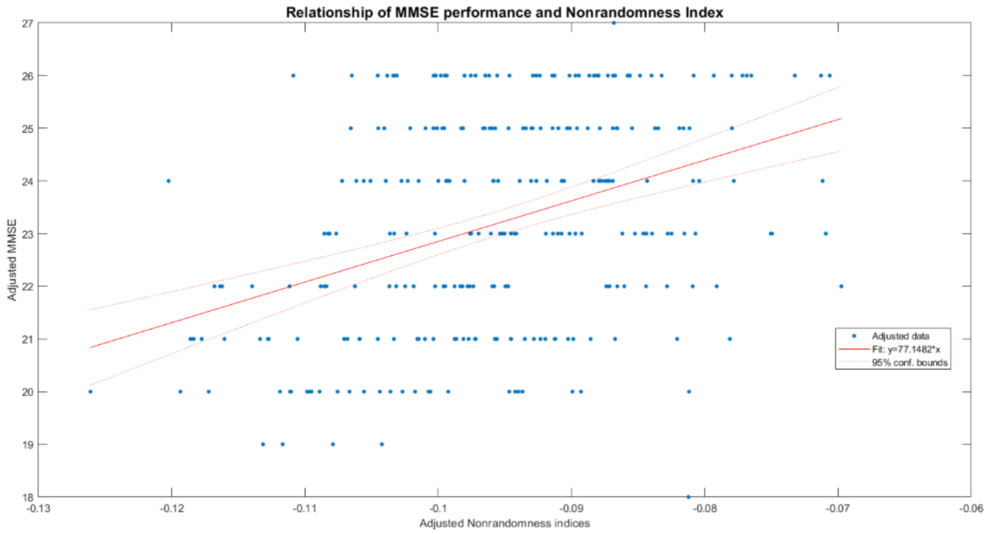

Supplementary Figure 3 Relationship of MMSE performance and nonrandomness index. The scatter plot illustrates the data with a fitted curve and confidence bounds for stepwise regression. The dots represent the MMSE score and adjusted nonrandomness indices of five brain regions of an individual with AD.

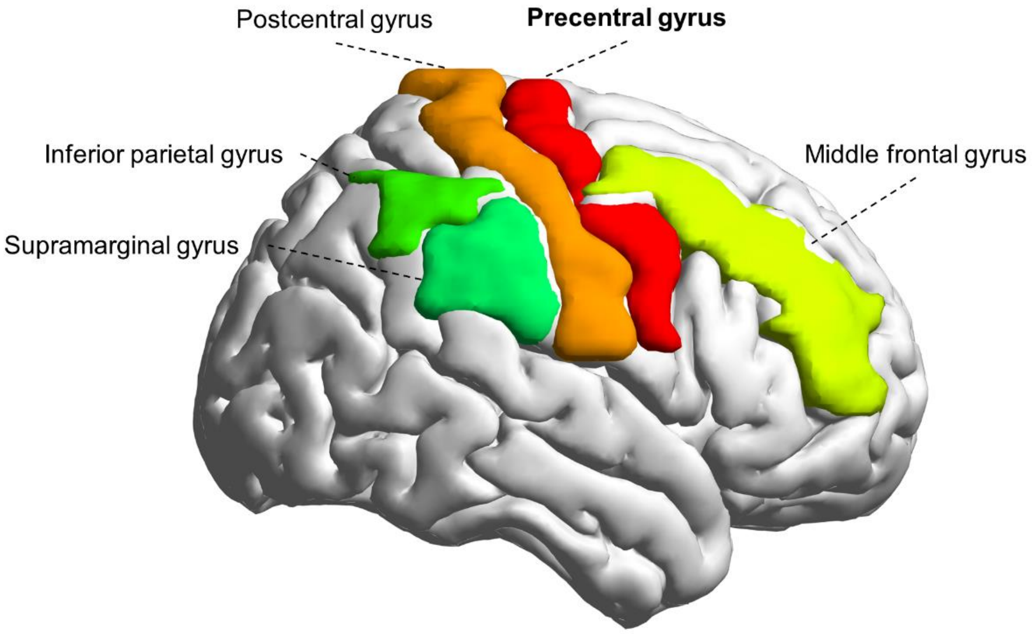

Supplementary Figure 4 Brain regions with structural similarity in cognitively normal older adults. The brain regions shown were found significantly similar in structural pattern to the right precentral gyrus. The morphological similarities to the right precentral gyrus ranked in order of the ipsilateral postcentral gyrus, middle frontal gyrus, inferior parietal gyrus, and supramarginal gyrus. These brain regions with morphological similarity present a tendency in spatial proximity.

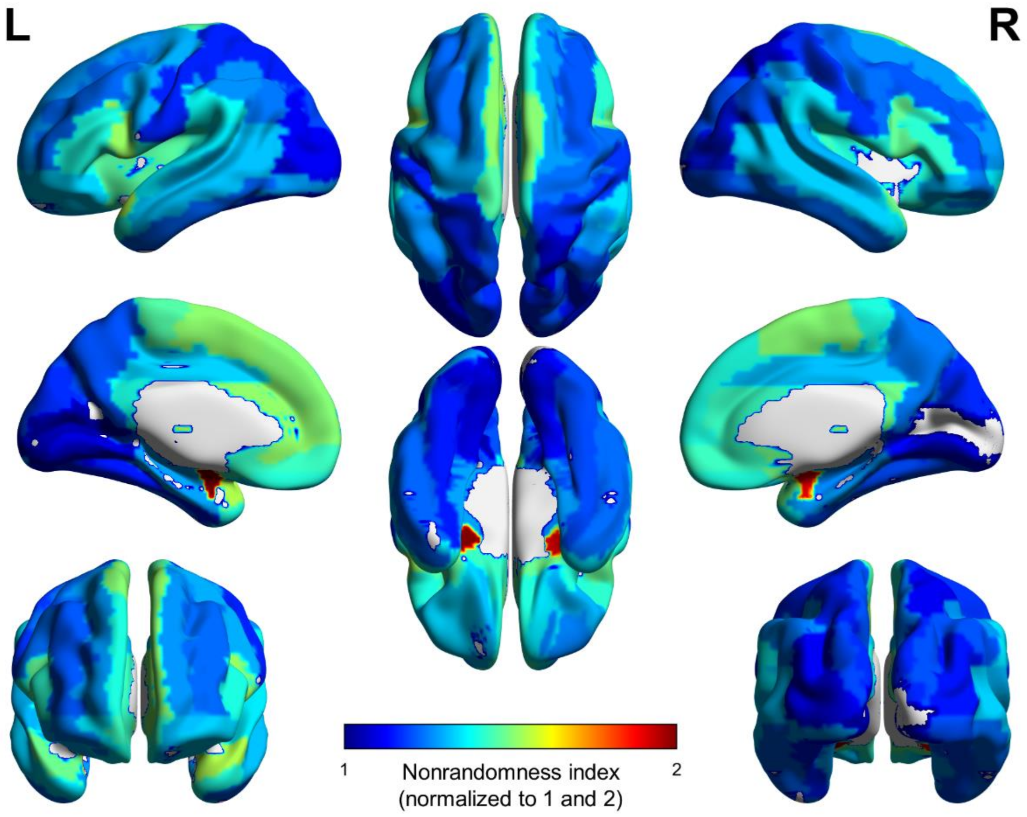

Supplementary Figure 5 The trend in structural nonrandomness in cognitively normal older adults. We computed the nonrandomness index of each brain region in cognitively normal older adults. The colors illustrate the group average, normalized nonrandomness indices in brain regions.

Supplementary Table 1 Regional structural similarities between AD and CN groups

AAL	Region	Distance
1	Precentral gyrus	0.014
2	Precentral gyrus	0.014
3	Superior frontal gyrus, dorsolateral	0.022
4	Superior frontal gyrus, dorsolateral	0.018
5	Superior frontal gyrus, orbital part	0.022
6	Superior frontal gyrus, orbital part	0.027
7	Middle frontal gyrus	0.026
8	Middle frontal gyrus	0.018
9	Middle frontal gyrus, orbital part	0.017
10	Middle frontal gyrus, orbital part	0.013
11	Inferior frontal gyrus, opercular part	0.021
12	Inferior frontal gyrus, opercular part	0.019
13	Inferior frontal gyrus, triangular part	0.024
14	Inferior frontal gyrus, triangular part	0.014
15	Inferior frontal gyrus, orbital part	0.025
16	Inferior frontal gyrus, orbital part	0.020
17	Rolandic operculum	0.022
18	Rolandic operculum	0.024
19	Supplementary motor area	0.023
20	Supplementary motor area	0.017
21	Olfactory cortex	**0.052***
22	Olfactory cortex	**0.044***
23	Superior frontal gyrus, medial	0.030
24	Superior frontal gyrus, medial	0.020
25	Superior frontal gyrus, medial orbital	0.021
26	Superior frontal gyrus, medial orbital	0.021
27	Gyrus rectus	0.028
28	Gyrus rectus	0.037
29	Insula	0.029
30	Insula	0.023
31	Anterior cingulate and paracingulate gyri	0.029
32	Anterior cingulate and paracingulate gyri	**0.038***
33	Median cingulate and paracingulate gyri	0.032
34	Median cingulate and paracingulate gyri	0.038
35	Posterior cingulate gyrus	**0.054***

36	Posterior cingulate gyrus	**0.039***
37	Hippocampus	**0.048***
38	Hippocampus	**0.050***
39	Parahippocampal gyrus	0.029
40	Parahippocampal gyrus	0.024
41	Amygdala	0.035
42	Amygdala	0.031
43	Calcarine fissure and surrounding cortex	0.019
44	Calcarine fissure and surrounding cortex	0.022
45	Cuneus	0.019
46	Cuneus	0.021
47	Lingual gyrus	0.021
48	Lingual gyrus	0.025
49	Superior occipital gyrus	0.015
50	Superior occipital gyrus	0.015
51	Middle occipital gyrus	0.022
52	Middle occipital gyrus	0.017
53	Inferior occipital gyrus	0.025
54	Inferior occipital gyrus	0.012
55	Fusiform gyrus	0.020
56	Fusiform gyrus	0.018
57	Postcentral gyrus	0.016
58	Postcentral gyrus	0.019
59	Superior parietal gyrus	0.019
60	Superior parietal gyrus	0.016
61	Inferior parietal, but supramarginal and angular gyri	0.022
62	Inferior parietal, but supramarginal and angular gyri	0.016
63	Supramarginal gyrus	0.021
64	Supramarginal gyrus	0.012
65	Angular gyrus	0.021
66	Angular gyrus	0.019
67	Precuneus	0.018
68	Precuneus	0.015
69	Paracentral lobule	0.012
70	Paracentral lobule	0.017
71	Caudate nucleus	0.020
72	Caudate nucleus	0.032

73	Lenticular nucleus, putamen	0.022
74	Lenticular nucleus, putamen	0.019
75	Lenticular nucleus, pallidum	0.016
76	Lenticular nucleus, pallidum	0.017
77	Thalamus	0.019
78	Thalamus	0.024
79	Heschl gyrus	0.026
80	Heschl gyrus	0.031
81	Superior temporal gyrus	0.017
82	Superior temporal gyrus	0.015
83	Temporal pole: superior temporal gyrus	0.028
84	Temporal pole: superior temporal gyrus	0.015
85	Middle temporal gyrus	0.016
86	Middle temporal gyrus	0.020
87	Temporal pole: middle temporal gyrus	0.022
88	Temporal pole: middle temporal gyrus	0.020
89	Inferior temporal gyrus	0.021
90	Inferior temporal gyrus	0.019

*significant level = 0.05 (right-tailed)

The odd AAL numbers represent the left side and the even numbers represent the right side of the brain. AAL: Automated Anatomical Labeling.

Supplementary Table 2 Significant group differences in inter-regional structural similarity in individuals with AD

AAL	Region	The number of regions	Regions with increased distance	The number of regions	Regions with decreased distance
1	Precentral gyrus	4	17, 21, 29, 30	-	-
2	Precentral gyrus	1	29	-	-
3	Superior frontal gyrus, dorsolateral	2	29, 30	-	-
4	Superior frontal gyrus, dorsolateral	3	21, 29, 37	-	-
5	Superior frontal gyrus, orbital part	-	-	-	-
6	Superior frontal gyrus, orbital part	-	-	-	-
7	Middle frontal gyrus	1	30	-	-
8	Middle frontal gyrus	1	29	-	-
9	Middle frontal gyrus, orbital part	-	-	-	-
10	Middle frontal gyrus, orbital part	-	-	-	-
11	Inferior frontal gyrus, opercular part	-	-	-	-
12	Inferior frontal gyrus, opercular part	-	-	-	-
13	Inferior frontal gyrus, triangular part	-	-	-	-
14	Inferior frontal gyrus, triangular part	-	-	-	-
15	Inferior frontal gyrus, orbital part	-	-	-	-
16	Inferior frontal gyrus, orbital part	-	-	-	-
17	Rolandic operculum	3	1, 19, 23	-	-
18	Rolandic operculum	-	-	-	-
19	Supplementary motor area	2	17, 29	-	-
20	Supplementary motor area	2	21, 30	-	-
21	Olfactory cortex	6	1, 4, 20, 24, 57, 61	-	-
22	Olfactory cortex	1	23	-	-
23	Superior frontal gyrus, medial	4	17, 22, 29, 48	-	-
24	Superior frontal gyrus, medial	4	21, 30, 54, 86	-	-
25	Superior frontal gyrus, medial orbital	1	29	-	-
26	Superior frontal gyrus, medial orbital	-	-	-	-
27	Gyrus rectus	-	-	-	-
28	Gyrus rectus	-	-	-	-
29	Insula	14	1, 2, 3, 4, 8, 19, 23, 25, 33, 52, 58, 62, 64, 71	-	-
30	Insula	7	1, 3, 7, 20, 24, 57, 84	-	-
31	Anterior cingulate and paracingulate gyri	-	-	-	-
32	Anterior cingulate and paracingulate gyri	-	-	-	-

33	Median cingulate and paracingulate gyri	1	29	1	38
34	Median cingulate and paracingulate gyri	-	-	1	38
35	Posterior cingulate gyrus	-	-	-	-
36	Posterior cingulate gyrus	-	-	-	-
37	Hippocampus	4	4, 71, 73, 83	8	39, 43, 47, 51, 55, 81, 84, 86
38	Hippocampus	2	72, 84	11	33, 34, 40, 50, 52, 55, 79, 80, 81, 83, 85
39	Parahippocampal gyrus	1	42	1	37
40	Parahippocampal gyrus	-	-	1	38
41	Amygdala	-	-	1	79
42	Amygdala	3	39, 78, 84	2	77, 80
43	Calcarine fissure and surrounding cortex	-	-	1	37
44	Calcarine fissure and surrounding cortex	-	-	-	-
45	Cuneus	-	-	-	-
46	Cuneus	-	-	-	-
47	Lingual gyrus	-	-	1	37
48	Lingual gyrus	1	23	-	-
49	Superior occipital gyrus	-	-	-	-
50	Superior occipital gyrus	-	-	1	38
51	Middle occipital gyrus	-	-	1	37
52	Middle occipital gyrus	1	29	1	38
53	Inferior occipital gyrus	-	-	-	-
54	Inferior occipital gyrus	1	24	-	-
55	Fusiform gyrus	-	-	2	37, 38
56	Fusiform gyrus	-	-	-	-
57	Postcentral gyrus	3	21, 30, 86	-	-
58	Postcentral gyrus	3	29, 85, 89	-	-
59	Superior parietal gyrus	-	-	-	-
60	Superior parietal gyrus	-	-	-	-
61	Inferior parietal, but supramarginal and angular gyri	1	21	-	-
62	Inferior parietal, but supramarginal and angular gyri	1	29	-	-
63	Supramarginal gyrus	-	-	-	-
64	Supramarginal gyrus	1	29	-	-
65	Angular gyrus	-	-	-	-
66	Angular gyrus	-	-	-	-
67	Precuneus	-	-	-	-

68	Precuneus	-	-	-	-
69	Paracentral lobule	-	-	-	-
70	Paracentral lobule	-	-	-	-
71	Caudate nucleus	2	29, 37	-	-
72	Caudate nucleus	1	38	-	-
73	Lenticular nucleus, putamen	1	37	-	-
74	Lenticular nucleus, putamen	-	-	-	-
75	Lenticular nucleus, pallidum	-	-	1	83
76	Lenticular nucleus, pallidum	-	-	-	-
77	Thalamus	-	-	1	42
78	Thalamus	1	42	-	-
79	Heschl gyrus	-	-	2	38, 41
80	Heschl gyrus	-	-	2	38, 42
81	Superior temporal gyrus	-	-	2	37, 38
82	Superior temporal gyrus	-	-	-	-
83	Temporal pole: superior temporal gyrus	1	37	2	38, 75
84	Temporal pole: superior temporal gyrus	3	30, 38, 42	1	37
85	Middle temporal gyrus	1	58	1	38
86	Middle temporal gyrus	2	24, 57	1	37
87	Temporal pole: middle temporal gyrus	-	-	-	-
88	Temporal pole: middle temporal gyrus	-	-	-	-
89	Inferior temporal gyrus	1	58	-	-
90	Inferior temporal gyrus	-	-	-	-

The pairs of brain regions had a p value below 10^{-12}. Brain regions are presented by AAL numbers. The odd AAL numbers represent the left side and the even numbers represent the right side of the brain. AAL: Automated Anatomical Labeling.

Supplementary Table 3 Group differences in nonrandomness indices

AAL	Region	T	P
1	Precentral gyrus	-3.28	0.001
2	Precentral gyrus	-1.74	0.082
3	Superior frontal gyrus, dorsolateral	-4.41	**< 0.001***
4	Superior frontal gyrus, dorsolateral	-3.56	**< 0.001***
5	Superior frontal gyrus, orbital part	1.75	0.081
6	Superior frontal gyrus, orbital part	-0.04	0.967
7	Middle frontal gyrus	-1.95	0.051
8	Middle frontal gyrus	-2.06	0.040
9	Middle frontal gyrus, orbital part	-1.24	0.214
10	Middle frontal gyrus, orbital part	-1.78	0.075
11	Inferior frontal gyrus, opercular part	-2.13	0.034
12	Inferior frontal gyrus, opercular part	-0.31	0.758
13	Inferior frontal gyrus, triangular part	-1.16	0.248
14	Inferior frontal gyrus, triangular part	-0.92	0.360
15	Inferior frontal gyrus, orbital part	-1.56	0.120
16	Inferior frontal gyrus, orbital part	-1.62	0.105
17	Rolandic operculum	5.24	**< 0.001***
18	Rolandic operculum	2.73	0.007
19	Supplementary motor area	-2.01	0.044
20	Supplementary motor area	-1.02	0.307
21	Olfactory cortex	2.04	0.041
22	Olfactory cortex	2.36	0.018
23	Superior frontal gyrus, medial	1.31	0.191
24	Superior frontal gyrus, medial	-1.03	0.304
25	Superior frontal gyrus, medial orbital	3.31	0.001
26	Superior frontal gyrus, medial orbital	3.14	0.002
27	Gyrus rectus	0.68	0.497
28	Gyrus rectus	1.37	0.172
29	Insula	1.46	0.145
30	Insula	7.62	**< 0.001***
31	Anterior cingulate and paracingulate gyri	3.55	**< 0.001***
32	Anterior cingulate and paracingulate gyri	6.17	**< 0.001***
33	Median cingulate and paracingulate gyri	3.57	**< 0.001***
34	Median cingulate and paracingulate gyri	4.24	**< 0.001***
35	Posterior cingulate gyrus	0.15	0.880

36	Posterior cingulate gyrus	-2.07	0.039
37	Hippocampus	6.82	**< 0.001***
38	Hippocampus	5.59	**< 0.001***
39	Parahippocampal gyrus	-0.56	0.576
40	Parahippocampal gyrus	-3.30	0.001
41	Amygdala	3.26	0.001
42	Amygdala	2.14	0.032
43	Calcarine fissure and surrounding cortex	3.95	**< 0.001***
44	Calcarine fissure and surrounding cortex	0.92	0.358
45	Cuneus	-0.53	0.597
46	Cuneus	-0.22	0.824
47	Lingual gyrus	-3.54	**< 0.001***
48	Lingual gyrus	-1.98	0.048
49	Superior occipital gyrus	-4.41	**< 0.001***
50	Superior occipital gyrus	-3.29	0.001
51	Middle occipital gyrus	-2.66	0.008
52	Middle occipital gyrus	-2.03	0.043
53	Inferior occipital gyrus	-5.01	**< 0.001***
54	Inferior occipital gyrus	-3.18	0.002
55	Fusiform gyrus	-1.35	0.178
56	Fusiform gyrus	-3.37	0.001
57	Postcentral gyrus	-1.64	0.102
58	Postcentral gyrus	-1.75	0.080
59	Superior parietal gyrus	-0.64	0.520
60	Superior parietal gyrus	-2.02	0.044
61	Inferior parietal, but supramarginal and angular gyri	0.02	0.984
62	Inferior parietal, but supramarginal and angular gyri	0.97	0.334
63	Supramarginal gyrus	4.15	**< 0.001***
64	Supramarginal gyrus	0.36	0.717
65	Angular gyrus	-1.70	0.090
66	Angular gyrus	-2.01	0.045
67	Precuneus	1.79	0.074
68	Precuneus	0.01	0.994
69	Paracentral lobule	0.94	0.349
70	Paracentral lobule	2.71	0.007
71	Caudate nucleus	-4.98	**< 0.001***
72	Caudate nucleus	-1.94	0.053

73	Lenticular nucleus, putamen	1.03	0.302
74	Lenticular nucleus, putamen	0.01	0.991
75	Lenticular nucleus, pallidum	-0.45	0.651
76	Lenticular nucleus, pallidum	1.29	0.198
77	Thalamus	1.56	0.119
78	Thalamus	3.17	0.002
79	Heschl gyrus	5.68	**< 0.001***
80	Heschl gyrus	2.97	0.003
81	Superior temporal gyrus	4.34	**< 0.001***
82	Superior temporal gyrus	3.08	0.002
83	Temporal pole: superior temporal gyrus	5.73	**< 0.001***
84	Temporal pole: superior temporal gyrus	4.88	**< 0.001***
85	Middle temporal gyrus	-1.35	0.179
86	Middle temporal gyrus	-1.52	0.129
87	Temporal pole: middle temporal gyrus	3.12	0.002
88	Temporal pole: middle temporal gyrus	0.92	0.359
89	Inferior temporal gyrus	-0.50	0.619
90	Inferior temporal gyrus	-3.16	0.002

*$p < 5.56 \times 10^{-4}$ (Bonferroni corrected)

The odd AAL numbers represent the left side and the even numbers represent the right side of the brain.

AAL: Automated Anatomical Labeling.

Supplementary Table 4 Association of structural randomness with cognitive performance in individuals with AD

AAL	Region	P (linear regression)	P (stepwise regression)
1	Precentral gyrus	0.508	-
2	Precentral gyrus	0.218	-
3	Superior frontal gyrus, dorsolateral	0.791	-
4	Superior frontal gyrus, dorsolateral	0.881	-
5	Superior frontal gyrus, orbital part	0.500	-
6	Superior frontal gyrus, orbital part	0.517	-
7	Middle frontal gyrus	0.812	-
8	Middle frontal gyrus	0.378	-
9	Middle frontal gyrus, orbital part	0.861	-
10	Middle frontal gyrus, orbital part	0.782	-
11	Inferior frontal gyrus, opercular part	0.820	-
12	Inferior frontal gyrus, opercular part	0.536	-
13	Inferior frontal gyrus, triangular part	0.188	-
14	Inferior frontal gyrus, triangular part	0.950	-
15	Inferior frontal gyrus, orbital part	0.188	-
16	Inferior frontal gyrus, orbital part	0.900	-
17	Rolandic operculum	0.174	-
18	Rolandic operculum	**0.025***	-
19	Supplementary motor area	0.164	-
20	Supplementary motor area	0.577	-
21	Olfactory cortex	0.111	-
22	Olfactory cortex	0.673	-
23	Superior frontal gyrus, medial	0.107	-
24	Superior frontal gyrus, medial	0.589	-
25	Superior frontal gyrus, medial orbital	0.417	-
26	Superior frontal gyrus, medial orbital	**0.089***	-
27	Gyrus rectus	0.196	-
28	Gyrus rectus	0.375	-
29	Insula	**0.022****	-
30	Insula	0.135	-
31	Anterior cingulate and paracingulate gyri	**0.093***	-
32	Anterior cingulate and paracingulate gyri	**0.011****	**0.009****
33	Median cingulate and paracingulate gyri	0.547	-
34	Median cingulate and paracingulate gyri	0.171	-

35	Posterior cingulate gyrus	0.115	-
36	Posterior cingulate gyrus	0.827	-
37	Hippocampus	0.580	-
38	Hippocampus	**0.005****	**0.011****
39	Parahippocampal gyrus	0.485	-
40	Parahippocampal gyrus	0.299	-
41	Amygdala	0.630	-
42	Amygdala	0.349	-
43	Calcarine fissure and surrounding cortex	**0.023****	-
44	Calcarine fissure and surrounding cortex	0.699	-
45	Cuneus	0.797	-
46	Cuneus	0.552	-
47	Lingual gyrus	0.831	-
48	Lingual gyrus	0.784	-
49	Superior occipital gyrus	0.778	-
50	Superior occipital gyrus	0.412	-
51	Middle occipital gyrus	0.510	-
52	Middle occipital gyrus	0.807	-
53	Inferior occipital gyrus	0.438	-
54	Inferior occipital gyrus	0.467	-
55	Fusiform gyrus	0.718	-
56	Fusiform gyrus	0.676	-
57	Postcentral gyrus	0.910	-
58	Postcentral gyrus	0.139	-
59	Superior parietal gyrus	0.514	-
60	Superior parietal gyrus	**0.021****	-
61	Inferior parietal, but supramarginal and angular gyri	0.074	-
62	Inferior parietal, but supramarginal and angular gyri	**0.071***	-
63	Supramarginal gyrus	0.429	-
64	Supramarginal gyrus	**0.030****	**<0.001*****
65	Angular gyrus	**0.014****	**0.003****
66	Angular gyrus	0.626	-
67	Precuneus	0.940	-
68	Precuneus	0.399	-
69	Paracentral lobule	0.553	-
70	Paracentral lobule	**0.030****	-
71	Caudate nucleus	**0.074***	-

72	Caudate nucleus	0.203	-
73	Lenticular nucleus, putamen	0.557	-
74	Lenticular nucleus, putamen	0.836	-
75	Lenticular nucleus, pallidum	0.363	-
76	Lenticular nucleus, pallidum	0.269	-
77	Thalamus	0.476	-
78	Thalamus	0.811	-
79	Heschl gyrus	**0.098***	-
80	Heschl gyrus	0.824	-
81	Superior temporal gyrus	0.909	-
82	Superior temporal gyrus	0.428	-
83	Temporal pole: superior temporal gyrus	0.900	-
84	Temporal pole: superior temporal gyrus	0.121	-
85	Middle temporal gyrus	**0.046****	**<0.001*****
86	Middle temporal gyrus	0.480	-
87	Temporal pole: middle temporal gyrus	0.380	-
88	Temporal pole: middle temporal gyrus	0.726	-
89	Inferior temporal gyrus	0.118	-
90	Inferior temporal gyrus	0.532	-

* $p < 0.1$, ** $p < 0.05$, *** $p < 0.001$

The odd AAL numbers represent the left side and the even numbers represent the right side of the brain.

AAL: Automated Anatomical Labeling.

Supplementary Table 5 Brain regions with significantly small or large IBS distances in CN group

AAL	Region	The number of regions	Regions with small IBS distance	The number of regions	Regions with large IBS distance
1	Precentral gyrus	4	7, 20, 57, 61	1	75
2	Precentral gyrus	4	8, 58, 62, 64	1	76
3	Superior frontal gyrus, dorsolateral	1	7	2	75, 76
4	Superior frontal gyrus, dorsolateral	1	8	1	75
5	Superior frontal gyrus, orbital part	-	-	4	75, 76, 78, 79
6	Superior frontal gyrus, orbital part	-	-	3	75, 76, 80
7	Middle frontal gyrus	4	1, 3, 20, 24	1	75
8	Middle frontal gyrus	4	2, 4, 19, 58	1	76
9	Middle frontal gyrus, orbital part	-	-	2	75, 76
10	Middle frontal gyrus, orbital part	-	-	4	37, 41, 75, 76
11	Inferior frontal gyrus, opercular part	-	-	1	75
12	Inferior frontal gyrus, opercular part	-	-	1	76
13	Inferior frontal gyrus, triangular part	1	85	1	75
14	Inferior frontal gyrus, triangular part	1	86	2	75, 76
15	Inferior frontal gyrus, orbital part	-	-	2	42, 75
16	Inferior frontal gyrus, orbital part	1	90	2	41, 76
17	Rolandic operculum	-	-	1	75
18	Rolandic operculum	-	-	2	75, 76
19	Supplementary motor area	1	8	1	76
20	Supplementary motor area	2	1, 7	1	75
21	Olfactory cortex	-	-	2	75, 76
22	Olfactory cortex	-	-	2	75, 76
23	Superior frontal gyrus, medial	-	-	2	75, 76
24	Superior frontal gyrus, medial	1	7	1	75
25	Superior frontal gyrus, medial orbital	-	-	3	42, 75, 76
26	Superior frontal gyrus, medial orbital	-	-	3	75, 76, 79
27	Gyrus rectus	-	-	4	41, 42, 75, 76
28	Gyrus rectus	-	-	2	75, 76
29	Insula	-	-	1	75
30	Insula	-	-	1	76
31	Anterior cingulate and paracingulate gyri	-	-	2	75, 76
32	Anterior cingulate and paracingulate gyri	-	-	-	-
33	Median cingulate and paracingulate gyri	-	-	2	75, 76

34	Median cingulate and paracingulate gyri	-	-	9	38, 39, 41, 42, 71, 75, 76, 78, 79
35	Posterior cingulate gyrus	-	-	3	37, 75, 76
36	Posterior cingulate gyrus	-	-	3	10, 36, 75
37	Hippocampus	-	-	2	35, 76
38	Hippocampus	-	-	4	35, 75, 76, 77
39	Parahippocampal gyrus	-	-	2	75, 76
40	Parahippocampal gyrus	-	-	7	10, 16, 27, 35, 54, 75, 76
41	Amygdala	-	-	5	15, 25, 27, 35, 76
42	Amygdala	-	-	-	-
43	Calcarine fissure and surrounding cortex	-	-	1	76
44	Calcarine fissure and surrounding cortex	1	67	-	-
45	Cuneus	-	-	-	-
46	Cuneus	-	-	2	75, 77
47	Lingual gyrus	-	-	3	75, 76, 78
48	Lingual gyrus	1	59	-	-
49	Superior occipital gyrus	-	-	1	76
50	Superior occipital gyrus	-	-	9	38, 39, 41, 42, 71, 75, 76, 78, 79
51	Middle occipital gyrus	-	-	-	-
52	Middle occipital gyrus	-	-	1	76
53	Inferior occipital gyrus	-	-	1	75
54	Inferior occipital gyrus	-	-	3	41, 76, 78
55	Fusiform gyrus	1	56	2	75, 76
56	Fusiform gyrus	1	55	2	75, 76
57	Postcentral gyrus	2	1, 61	1	75
58	Postcentral gyrus	5	2, 8, 60, 62, 64	1	76
59	Superior parietal gyrus	4	49, 61, 67, 68	-	-
60	Superior parietal gyrus	2	58, 67	-	-
61	Inferior parietal, but supramarginal and angular gyri	3	1, 57, 59	1	75
62	Inferior parietal, but supramarginal and angular gyri	2	2, 58	1	76
63	Supramarginal gyrus	-	-	1	75
64	Supramarginal gyrus	4	2, 58, 82, 86	1	76
65	Angular gyrus	-	-	1	75
66	Angular gyrus	-	-	1	76
67	Precuneus	4	45, 59, 60, 68	-	-

68	Precuneus	2	59, 67	-	-
69	Paracentral lobule	-	-	-	-
70	Paracentral lobule	-	-	1	75
71	Caudate nucleus	-	-	2	35, 76
72	Caudate nucleus	-	-	3	79, 83, 87
73	Lenticular nucleus, putamen	-	-	-	-
74	Lenticular nucleus, putamen	-	-	1	75
75	Lenticular nucleus, pallidum	-	-	56	1, 3, 4, 5, 6, 7, 9, 10, 11, 13, 14, 15, 17, 18, 20, 21, 22, 23, 24, 25, 26, 27, 28, 29, 31, 33, 34, 35, 36, 37, 39, 40, 41, 47, 48, 53, 55, 56, 57, 61, 63, 65, 70, 74, 76, 77, 79, 80, 81, 82, 83, 84, 85, 87, 88, 89
76	Lenticular nucleus, pallidum	-	-	52	2, 3, 5, 6, 8, 9, 10, 12, 14, 16, 18, 19, 21, 22, 23, 25, 26, 27, 28, 30, 31, 33, 35, 36, 38, 39, 40, 41, 42, 44, 48, 50, 52, 54, 55, 56, 58, 62, 64, 66, 71, 75, 77, 79, 80, 82, 83, 84, 86, 87, 88, 90
77	Thalamus	-	-	7	39, 47, 75, 76, 78, 79, 84
78	Thalamus	-	-	6	5, 35, 48, 54, 77, 80
79	Heschl gyrus	-	-	7	5, 26, 35, 72, 75, 76, 77
80	Heschl gyrus	-	-	4	6, 75, 76, 78
81	Superior temporal gyrus	1	85	1	75
82	Superior temporal gyrus	2	64, 86	2	75, 76
83	Temporal pole: superior temporal gyrus	-	-	3	72, 75, 76
84	Temporal pole: superior temporal gyrus	-	-	3	75, 76, 77
85	Middle temporal gyrus	2	13, 81	1	75
86	Middle temporal gyrus	3	14, 64, 82	1	76

87	Temporal pole: middle temporal gyrus	-	-	3	72, 75, 76
88	Temporal pole: middle temporal gyrus	-	-	2	75, 76
89	Inferior temporal gyrus	-	-	1	75
90	Inferior temporal gyrus	1	16	1	76

The pairs of brain regions had a *p* value below 0.05 (two-tailed uncorrected). Brain regions are presented by AAL numbers. The odd AAL numbers represent the left side and the even numbers represent the right side of the brain. AAL: Automated Anatomical Labeling.

Supplementary Table 6 Average nonrandomness indices in cognitively normal older adults

AAL	Region	Mean nonrandomness index	Normalized index
44	Calcarine fissure and surrounding cortex	**0.197***	1.000
43	Calcarine fissure and surrounding cortex	0.204	1.050
47	Lingual gyrus	0.210	1.094
60	Superior parietal gyrus	0.213	1.114
51	Middle occipital gyrus	0.213	1.116
48	Lingual gyrus	0.215	1.130
52	Middle occipital gyrus	0.217	1.145
55	Fusiform gyrus	0.217	1.149
59	Superior parietal gyrus	0.218	1.155
57	Postcentral gyrus	0.219	1.159
46	Cuneus	0.219	1.163
45	Cuneus	0.220	1.169
58	Postcentral gyrus	0.221	1.178
49	Superior occipital gyrus	0.224	1.196
56	Fusiform gyrus	0.224	1.199
54	Inferior occipital gyrus	0.225	1.202
50	Superior occipital gyrus	0.225	1.206
67	Precuneus	0.225	1.206
66	Angular gyrus	0.225	1.207
8	Middle frontal gyrus	0.226	1.211
89	Inferior temporal gyrus	0.228	1.226
7	Middle frontal gyrus	0.228	1.230
90	Inferior temporal gyrus	0.229	1.232
2	Precentral gyrus	0.229	1.236
65	Angular gyrus	0.230	1.244
68	Precuneus	0.231	1.249
10	Middle frontal gyrus, orbital part	0.232	1.254
76	Lenticular nucleus, pallidum	0.232	1.260
38	Hippocampus	0.232	1.260
61	Inferior parietal, but supramarginal and angular gyri	0.234	1.269
3	Superior frontal gyrus, dorsolateral	0.234	1.274
62	Inferior parietal, but supramarginal and angular gyri	0.235	1.278
40	Parahippocampal gyrus	0.235	1.279
4	Superior frontal gyrus, dorsolateral	0.235	1.279

53	Inferior occipital gyrus	0.236	1.284
39	Parahippocampal gyrus	0.236	1.288
37	Hippocampus	0.236	1.290
75	Lenticular nucleus, pallidum	0.237	1.293
1	Precentral gyrus	0.238	1.299
16	Inferior frontal gyrus, orbital part	0.238	1.304
82	Superior temporal gyrus	0.239	1.306
5	Superior frontal gyrus, orbital part	0.240	1.314
34	Median cingulate and paracingulate gyri	0.240	1.314
85	Middle temporal gyrus	0.240	1.317
86	Middle temporal gyrus	0.242	1.330
9	Middle frontal gyrus, orbital part	0.242	1.332
33	Median cingulate and paracingulate gyri	0.242	1.332
15	Inferior frontal gyrus, orbital part	0.243	1.335
64	Supramarginal gyrus	0.245	1.350
88	Temporal pole: middle temporal gyrus	0.245	1.352
6	Superior frontal gyrus, orbital part	0.245	1.352
81	Superior temporal gyrus	0.246	1.359
63	Supramarginal gyrus	0.249	1.383
35	Posterior cingulate gyrus	0.252	1.402
18	Rolandic operculum	0.252	1.403
13	Inferior frontal gyrus, triangular part	0.252	1.404
24	Superior frontal gyrus, medial	0.252	1.405
70	Paracentral lobule	0.252	1.405
87	Temporal pole: middle temporal gyrus	0.252	1.405
28	Gyrus rectus	0.252	1.407
30	Insula	0.253	1.412
27	Gyrus rectus	0.253	1.412
17	Rolandic operculum	0.253	1.413
14	Inferior frontal gyrus, triangular part	0.254	1.420
71	Caudate nucleus	0.254	1.421
79	Heschl gyrus	0.255	1.428
21	Olfactory cortex	0.256	1.431
32	Anterior cingulate and paracingulate gyri	0.256	1.433
25	Superior frontal gyrus, medial orbital	0.256	1.434
26	Superior frontal gyrus, medial orbital	0.256	1.435
80	Heschl gyrus	0.257	1.440

69	Paracentral lobule	0.259	1.459
29	Insula	0.260	1.463
74	Lenticular nucleus, putamen	0.261	1.468
12	Inferior frontal gyrus, opercular part	0.262	1.475
84	Temporal pole: superior temporal gyrus	0.263	1.483
72	Caudate nucleus	0.263	1.486
22	Olfactory cortex	0.263	1.488
36	Posterior cingulate gyrus	0.263	1.488
78	Thalamus	0.264	1.491
23	Superior frontal gyrus, medial	0.266	1.506
20	Supplementary motor area	0.266	1.507
73	Lenticular nucleus, putamen	0.266	1.509
19	Supplementary motor area	0.267	1.514
31	Anterior cingulate and paracingulate gyri	0.267	1.517
11	Inferior frontal gyrus, opercular part	0.268	1.526
77	Thalamus	0.270	1.536
83	Temporal pole: superior temporal gyrus	0.271	1.547
42	Amygdala	**0.319***	1.899
41	Amygdala	**0.333***	2.000

*significant level = 0.05 (two-tailed)

The odd AAL numbers represent the left side and the even numbers represent the right side of the brain.

AAL: Automated Anatomical Labeling.
